# DNA methylation-based classification and identification of renal cell carcinoma prognosis-subgroups

**DOI:** 10.1186/s12935-019-0900-4

**Published:** 2019-07-16

**Authors:** Wenbiao Chen, Jia Zhuang, Peizhong Peter Wang, Jingjing Jiang, Chenhong Lin, Ping Zeng, Yan Liang, Xujun Zhang, Yong Dai, Hongyan Diao

**Affiliations:** 1 State Key Laboratory for Diagnosis and Treatment of Infectious Diseases, Collaborative Innovation Center for Diagnosis and Treatment of Infectious Diseases, The First Affiliated Hospital, College of Medicine, Zhejiang University, 79 Qing Chun Road, Hangzhou, China; 2 Clinical Medical Research Center, Shenzhen People’s Hospital, The Second Clinical Medical College of Jinan University, 1017 Dongmen North Road, Luohu District, Shenzhen, Guangdong China; 3 Department of Urinary Surgery, Puning People’s Hospital, Puning People’s Hospital Affiliated To Southern Medical University, 30 Liusha Avenue, Jieyang, Guangdong China; 4 Division of Community Health and Humanities, Faculty of Medicine, Memorial University of Newfoundland, St. John’s, Newfoundland Canada

**Keywords:** Renal cell carcinoma, DNA methylation, Prognosis subgroups, Molecular subtypes

## Abstract

**Background:**

Renal cell carcinoma (RCC) is the most common kidney cancer and includes several molecular and histological subtypes with different clinical characteristics. The combination of DNA methylation and gene expression data can improve the classification of tumor heterogeneity, by incorporating differences at the epigenetic level and clinical features.

**Methods:**

In this study, we identified the prognostic methylation and constructed specific prognosis-subgroups based on the DNA methylation spectrum of RCC from the TCGA database.

**Results:**

Significant differences in DNA methylation profiles among the seven subgroups were revealed by consistent clustering using 3389 CpGs that indicated that were significant differences in prognosis. The specific DNA methylation patterns reflected differentially in the clinical index, including TNM classification, pathological grade, clinical stage, and age. In addition, 437 CpGs corresponding to 477 genes of 151 samples were identified as specific hyper/hypomethylation sites for each specific subgroup. A total of 277 and 212 genes corresponding to DNA methylation at promoter sites were enriched in transcription factor of GKLF and RREB-1, respectively. Finally, Bayesian network classifier with specific methylation sites was constructed and was used to verify the test set of prognoses into DNA methylation subgroups, which was found to be consistent with the classification results of the train set. DNA methylation-based classification can be used to identify the distinct subtypes of renal cell carcinoma.

**Conclusions:**

This study shows that DNA methylation-based classification is highly relevant for future diagnosis and treatment of renal cell carcinoma as it identifies the prognostic value of each epigenetic subtype.

**Electronic supplementary material:**

The online version of this article (10.1186/s12935-019-0900-4) contains supplementary material, which is available to authorized users.

## Background

Renal cell carcinoma is the most lethal urologic malignancies and accounts for 3% of all malignant tumors. In recent decades, the incidence of RCC has been rising due to the advances in ultrasound, computed tomography and magnetic resonance imaging used for the early detection of renal tumors. Although recent molecular targeting therapy has improved the overall survival of RCC patients, long-term prognosis remains precarious [1, 2]. Previous, pathological assessments determined the risk of recurrence and differentiation in RCC patients, thus guiding the diagnosis and treatment of recurrent diseases. The classification and diagnostic criteria of RCC are indeterminate and have a high interobserver bias. As a result, 30% of patients develop metastatic disease after treatment, with a median survival period of 1 year [3]. There are growing pieces of evidence that even on the basis of the same RCC histological subtype, there is an inconsistent representation of sets of histologically and molecularly heterogeneous diseases [4]. With further understanding of the morphology, immunohistochemistry, molecular and epidemiological features of RCC, the World Health Organization (WHO) revised the classification of renal cell tumors in 2016. It mainly focuses on the molecular pathological features of renal cell tumors [5]. To this end, a more detailed and comprehensive understanding of the molecular classification characteristics of RCC is required.

Currently, the analysis of molecular characteristics has shown that the RCC has obvious specificity. Since molecular subtype supplements histopathology, invaluable insights can be drawn from integrated classifications, which could refine and impact the future application of precision-focused, personalized clinical management of renal cell carcinoma [6]. Ricketts et al. performed a comprehensive genomic and phenotypic analysis on RCC in order to identify the common and specific molecular characteristics and laid the foundation for the development of subtype-specific treatment and management strategies for these cancer patients [7]. Wu et al. used an unsupervised Consensus Clustering algorithm to identify three distinct molecular subtypes of clear renal clear cell carcinoma (the most common subtype) on the basis of hierarchical clustering. Subtypes with poor prognosis were irregularly up-regulated in focal adhesion and cytoskeleton-related pathways, and the expression of core genes, including *LIMK1*, *COL5A1*, *MMP9* and *CCL26*, were negatively associated with the prognosis of the patients [8]. To produce a characteristic biomarker to determine the high and low risk of clear renal cell carcinoma, Samira et al. developed a 34 genes subtype predictor that divided 54 patients with metastatic RCC into good and poor follow-up groups. A model containing subtype-inclusive model was established to analyze the survival outcome of the patients [9, 10].

DNA methylation, one of the most common epigenetic modifications, plays a crucial role in the regulation of the structure and expression of genes and is involved in a variety of biological processes. In addition, changes in DNA methylation are inextricably linked to all aspects of cancer genomics and have been shown to be closely related to changes in the gene expression, sequence and copy number [11]. In addition, DNA methylation often occurs on the C (cytosine) of 5′-CpG-3′ and as a result generates 5-methyldeoxycytidine (5mC). DNA methylation has become an important research topic in epigenetics and epigenomics because of the close relationship between aberrant DNA methylation and physio-pathologic mechanisms underlying an array of human diseases, especially CpG island methylation. CpG-rich regions (CpG islands) are found in about 40–50% of promoter regions of human genes. Abnormal CpG island hypermethylation of tumor suppressor genes and hypomethylation of oncogenes are vital in carcinogenesis [12, 13]. Therefore, as a promising molecular marker of RCC, abnormal DNA methylation appears in early detection, prognosis prediction, molecular classification and therapeutic targets [14]. Previous studies have shown that the increase in promoter hypermethylation frequency is associated with higher stage and grade in clear cell RCC, according to The Cancer Genome Atlas (TCGA) Research Network for cancer genome maps [15]. Ana et al. identified groups such as *OXR*, *MST1R* and *HOXA9* promoter methylation that positively identified renal cell tumors and differentiation of subtypes. In addition, the groups may improve risk stratification in patients with small renal masses, helping clinicians to determine the best treatment strategy [16]. Gabriel et al. found that the DNA methylation profiles of RCC subtypes can be divided into two main epigenetic clusters: one consists of clear-cell RCC, papillary RCC, mucinous and spindle cell carcinomas and translocation RCC; the other includes oncocytoma and chromophobe RCC [17]. Moreover, an epigenetic chart of the 56 genes that identify the ontogeny of renal cells can predict the results of clear cell RCC. However, their classification may not be detailed enough and has not yet been fully tested for specific sites related to each category.

In this study, our aim is to fully identify the entire DNA methylated profiles of RCC from the TCGA database in order to identify the biological and clinical-related subgroups. Our datasets and the accompanying classification schemes may help to identify new markers or molecular subtypes of RCC in order to accurately subdivide patients with renal cell carcinoma. Furthermore, the RCC classification based on integrated profiling combined with histology and DNA methylation profiling analysis provides a more accurate prediction of clinical behavior compared to the histology alone and provides higher risk assessment accuracy for individual patients. In addition to the identification of clinical-related groups and a new classification basis, our datasets can provide an in-depth understanding into the pathogenesis of RCC and guide accurate decision-making process during diagnosis and treatment.

## Methods

### Date preprocessing and DNA methylation loci in RCC

A total of 485 RCC samples containing DNA methylation data generated from the Illumina Infinium HumanMethylation450 Bead-Chip array of TCGA [18] were downloaded by UCSC Cancer Browser [19] on June 25, 2018. RCC samples with more than 30 days of follow-up were selected to match the methylation profile. Ultimately, 307 RCC samples were selected for methylation analysis. Methylation level of probes with missing data in more than 70% of the samples were removed. Based on the discovery of cross-reactive probes and polymorphic CpGs by the Illumina Infinium HumanMethylation450 microarray, CpG sites of cross-reactive probes in the genome were removed. The other unidentified probes were estimated using the k-nearest neighbor (KNN) imputation approach in the sva R package [20]. Unstable genomic sites, which contained CpGs in sex chromosomes and single nucleotide polymorphisms were discarded. Finally, 208,022 CpG sites were obtained for further analysis. This study was approved by the Clinical Research Ethics Committee of College of Medicine, Zhejiang University.

Next, 307 RCC samples were randomly divided into two groups, a train group and a test group. In both groups, the age distribution, clinical stage, follow-up time and mortality were similar. Finally, 307 RCC samples were divided into 153 experimental samples in train group and 154 verified samples in test group.

### Univariate COX model and multivariate COX proportional risk regression model analysis

To obtain a specific prognostic subgroup of molecular subtypes of RCC, CpG sits of DNA methylation that affect the survival were used as the classification feature. Firstly, based on the methylation level of each CpG site, the survival coxph function R package was used to establish a univariate COX proportional risk regression model. Next, significant CpG sites were selected for multivariate COX proportional risk regression model. Moreover, the TNM classification, pathological grading, and clinical staging were used as covariates to identify independent prognostic factors. For each CpG site, the formula for multivariate COX proportional risk regression model was:

$$ h(t,x)_{{\text{i}}}  = h0(t)\exp ( \upbeta_{{{\text{methylation}}}} {\text{methylation}}_{i}  +  \upbeta _{{{\text{TNM}}}} {\text{TNM}} + \upbeta_{{{\text{grade}}}} {\text{grade}} + \upbeta _{{{\text{stage}}}} {\text{stage}}) $$

where *h* (*t*, *x*)_i_ is a risk function associated with covariates at time *t* (TNM classification, pathological grade, clinical stage). Methylation_i_ is the vector of the level of CpG_i_ methylation in the sample, TNM, grade and stage represent their respective vector values. β stands for regression coefficient of methylation, TNM, grade, and stage. The *p* values of the Cox regression coefficients were calculated and corrected by multiple test correction using the method of Benjamini and Hochberg false discovery rate.

### Selection of molecular subtypes by consistent clustering

The K-means clustering algorithm in the ConcensusClusterPlus R packet [21] was used for consistent clustering to determine tumor subgroups based on the most variable CpG sites. The K-means clustering algorithm was designed to classify a pre-specified dataset into k clusters. This algorithm defined “consensus” clustering by measuring the stability of clustering results from a given clustering method applied to a random subset of data. At each iteration, 80% of the tumor samples were selected, the K-means algorithm and Euclidean square distance measure were used for groups of k ranging from 2 to 20. More than 100 iterations were carried out and the stability of each cluster was determined. The maximum number of clusters with at least 90% cluster consensus were chosen.

The optimal cluster number was determined by the cumulative distribution function and the delta area plot. We considered the optimal number of clusters should be that the consistency of the cluster was relatively high, the coefficient of variation was relatively low, and the area under the CDF curve did not increase significantly. The number of categories was selected based on the relative non-significant change in the area under CDF curve. The pheatmap R package was used to construct the consensus cluster heatmap.

### Analysis of the clinical characteristics of molecular subtypes

The Kaplan–Meier method was used to construct the overall survival curve for RCC subgroups defined by DNA methylation spectrum and the statistical differences between clusters were determined by the log-rank test. R Bioconductor survival package was used for survival analysis, and Chi square test was used to comprehensively analyze the association between clinical and biological characteristics of DNA methylation clusters.

### Identification of specific DNA methylation markers and transcription factor enrichment of specific methylation site annotated genes

To identify the molecular types of methylation-based RCC, the Epidiff software was applied to reveal specific CpG sites that defined specific DNA methylation. EpiDiff is an integrated software platform that provides bioinformatic analysis on quantitative differential chromatin modification region (QDCMR), quantitative differentially methylated region (QDMR), and quantitative differential expressed gene (QDEG). The free platform and free species nature of EpiDiff makes it suitable for unprecedented epigenome profiles described by high-throughput experimental techniques using microarrays and next-generation sequencing [22]. To identify specific hypermethylation or hypomethylation of DNA CpG sites within specific RCC subgroups, QDMR software (http://bioinfo.hrbmu.edu.cn/qdmr) was used to quantify methylation differences and identify differentially methylated regions in multiple RCC samples [23]. Next, the significant CpG sites across the classified subgroups were used to find specific CpGs in each subgroup. For each subgroup, the average DNA methylation level for each sample with significant CpGs was calculated and the matrix with 3389 × 6 dimensions was input into the QDMR. The threshold of standard deviation parameter was set to 0.04.

DNA methylation at the promoter site can promote or inhibit gene expression. To explore the mechanism of the specific CpG sites, the transcription factor (TF) enrichment analysis of the corresponding genes at the promoter region of specific CpG sites was carried out by g:Profiler. G:Profiler is a simple, user-friendly Web interface with powerful visualization abilities to capture gene ontologies, pathways, or transcription factor binding sites, or even individual gene levels [24]. The genes corresponding to the TF enrichment promoters were displayed by Cytoscape.

### Construction of Bayesian classifier and verification of test set data

In order to verify the distinguished ability of specific CpG sits, specific CpG sites which were identified by Epidiff software were constructed by Bayesian classifier. The samples in the test set were assigned to the corresponding subgroups using this classified model. The performance of the model was verified by tenfold cross-validation method, and the accuracy of the model was evaluated using accuracy rate. The pROC packet in R Program was used to establish a receiver Operation characteristic Curve (ROC). To verify the stability and reliability of the model, a prognostic model similar to the train group was used to assign a class label to 154 test samples.

## Results

### Characteristics of RCC samples evaluated for DNA methylation

The DNA methylation profiles of RCC samples from the TCGA database were used to cluster the RCC prognostic molecular subgroups. In total, 208,022 CpG sites were successfully screened for methylation in 307 RCC samples. For each of the CpG that sites in the train set, univariate Cox proportional hazards regression model was applied to each methylation site and the survival data recorded. 52,878 significant CpG sites were identified that could influence patient survival (*p *< 0.05). T (*p *= 1.32483e−06), N (*p *= 0.00095), M (*p *= 1.32483e−06), Stage (*p *= 1.787979e−06), and Grade (*p *= 6.581784e−07) were also found to be significant factors based on the univariate Cox proportional risk regression models analysis. Then, 52,878 significant CpG sites were introduced into the multivariate Cox proportional risk regression models, including TNM classification, pathological grade, clinical stage as covariates to investigate the independent prognostic factors. Finally, we got 3389 significant CpG sites for further prognosis subgroups analysis.

### Consensus clustering of DNA methylation of RCC identified prognosis subgroups

**Fig. 1:**
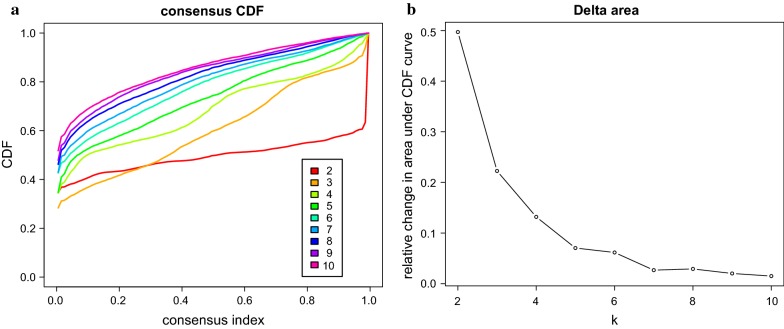
Consensus clustering of RCC distinct DNA methylation prognostic subgroups. **a** CDF curve. **b** CDF Delta area curve. Delta area curve of consensus clustering, indicating the relative change in area under the cumulative distribution function (CDF) curve for each category number k compared with k-1. The horizontal axis represents the category number k and the vertical axis represents the relative change in area under CDF curve

**Fig. 2:**
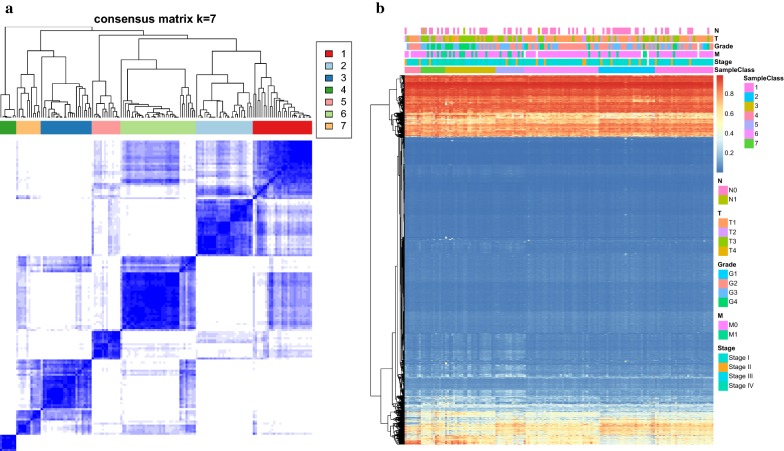
Cluster Analysis of 7 Molecular subtypes by DNA methylation classification. **a** The heatmap corresponding to the consensus matrix for 7 molecular subtypes obtained by applying consensus clustering. **b** The heatmap of 3389 CpG sites in 7 clusters

In order to screen distinct DNA methylation prognostic molecular subtypes of RCC, consistent clustering of 3389 significant CpG sites were constructed. Then, the average cluster consensus and the coefficient of variation among clusters were calculated for each category number to obtain the optimal number of classes. According to the CDF curve, the clustering result is relatively stable when the cluster is 7 or 8 (Fig. 1a). To further observe the CDF delta area curve, it can be seen that cluster has a stable clustering result when it is selected as 7, as the area under the CDF curve begins to stabilize after 7 categories (Fig. 1b). Thus, 7 was regarded as the optimal number of categories for further analysis. According to the result of consistent clustering, we selected the stable clustering result of 7 to construct a consensus matrix graph, which was a better visualization tool in the assessment of the composition of clustering and number. The color-coded heatmap is seen to correspond to the consensus matrix, 153 RCC samples were assigned to 7 categories. Additionally, a color-coded heatmap characterized by blue blocks along the diagonal on a white background revealed a well-defined 7-block structure (Fig. 2a). Further, the heatmaps that corresponded to 3389 CpG sites were generated by the heatmap function with DNA methylation classification with TNM classification [25], pathological grade [26], clinical stage [27], and age [28] as the annotations. It can be seen that the abundance of most CpG sites in each sample is low. However, there were obvious differences found in DNA methylation profiles among these 7 categories (Fig. 2b).

**Fig. 3:**
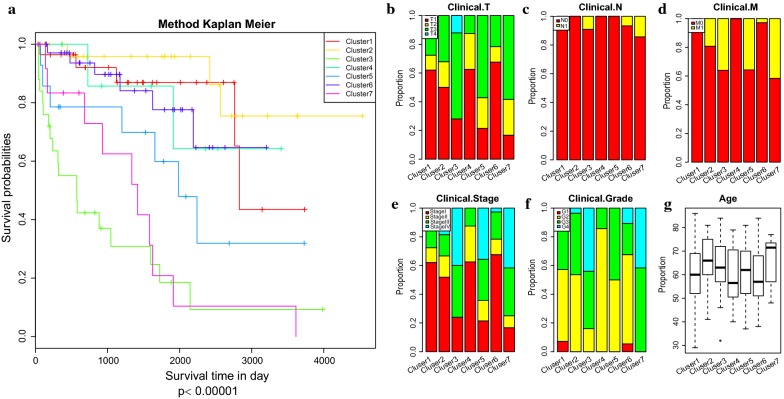
Characterization of different features of DNA methylation clustering. **a** Prognostic differences among the 7 clusters. **d** Proportion of different degrees of invasion in the 7 clusters. **c** Proportion of different degrees of lymphatic metastasis in the 7 clusters. **d** Proportion of different degrees of distant metastasis in the 7 clusters. **e** Proportion of clinical stage in the 7 clusters. **f** Proportion of pathological grade in the 7 clusters. **g** Age distribution in the 7 clusters

The Kaplan–Meier plot showed the survival of RCC defined by the methylation-based consensus clustering had a significant difference amount the 7 clusters (Fig. 3a). Among them, clusters 1 and 2 had the best prognosis, whereas clusters 3 and 7 had the worst prognosis. Understanding the TNM classification, pathological grade, clinical stage, and age can aid clinicians in choosing the best treatment for patients in their current condition and predict the prognosis. Thus, we analyzed the distribution of TNM classification, pathological grade, clinical stage, and age in the prognosis of 7 molecular subtypes (Fig. 3b–g). Some of the DNA methylation subgroups reflected differently in the TNM classification, pathological grade, clinical stage and age subtypes, whereas different DNA methylation subgroups were also found in the same TNM classification, pathological grade, clinical stage and age subtypes. These results demonstrated that patients in different TNM classification, pathological grade, clinical stage and age subtypes share the same DNA methylation characteristics and specific subtypes of TNM classification, pathological grade, clinical stage and that age contained different DNA methylation characteristics. Also, we found that the DNA methylation status represents a more elaborate classification analysis for RCC and also that DNA methylation can serve as a commendable biomarker for RCC classification. For example, clusters 1 and 6 had low invasiveness (Fig. 3b), clusters 3 and 7 had high relevance with lymphatic metastasis and distant metastasis (Fig. 3c, d), and clusters 3 and 7 connected with high pathological grade and high clinical stage (Fig. 3e, f). This gave a detailed description of the reason for the clusters 3 and 7 having the worst prognosis, as clusters 3 and 7 were more likely to metastasize and develop into a higher degree of malignancy, even though they had the same underlying aetiology, such as DNA methylation abnormalities. Also, it might explain the reason for the low TNM classification (such as T1 in cluster 3, primary tumor which is limited to kidney, maximum diameter ≤ 7 cm) still had higher degree of malignancy (such as G4 in cluster 3, tumor with low differentiation or no differentiation) and had higher chance of deterioration (such as stage 4 in cluster 3, tumor disease in terminal stage), as DNA methylation changes in carcinogenesis. These results indicate that these DNA methylation profiles can help to understand the etiology of RCC and, most importantly, they demonstrated clinically applicable biomarkers for use in the early detection of kidney cancer.

### Clinical and biological characteristics with DNA methylation clustering

Next, we investigated whether the DNA methylation clustering of RCC was relevant to clinical and biological characteristics. The clinical information of RCC was obtained from the TCGA Database. Then, we analyzed the clinical characteristics of tumor/normal, TNM classification, pathological grade, clinical stage, age, and gender in each group. The Chi square test was used to analyze the global associations between clinical and biological characteristics of DNA methylation clustering. Table 1 shows the tumor/normal, TNM classification, pathological grade, clinical stage, age was significantly different among the DNA methylation prognosis clusters (p < 0.05), but no significant difference was detected in gender (*p *> 0.05). This finding implied there was evidence of a strong association between the DNA methylation prognosis clustering and the clinical and biological characteristics.

**Table 1:** The associations between clinical and biological characteristics with DNA methylation clustering on Chi square test

Clinical attributes	Subclasses	P-value
Cancer_normal	Cancer	0.0003
	Normal	
T	T1	0.0019
	T2	
	T3	
	T4	
N	N0	0.0026
	N1	
M	M0	
	M1	
Stage	S1	0.0000
	S2	
	S3	
	S4	
Grade	G1	0.0000
	G2	
	G3	
	G4	
Age	Young (age < 60)	0.0292
	Old (age > 60)	
Sex	Male	0.1722
	Female	

### Identification of specific DNA methylation markers and corresponding genes

**Fig. 4:**
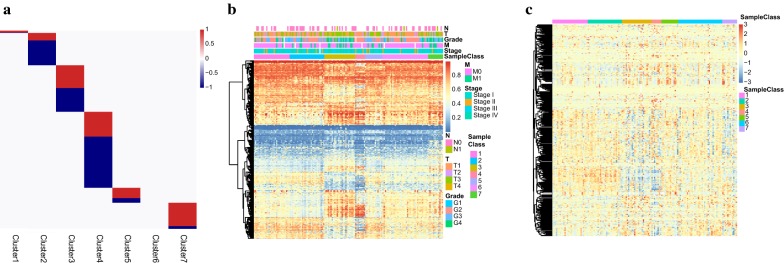
Specific hypermethylation/hypomethylation CpG sites and corresponding genes for each DNA methylation cluster. **a** Distribution of specific CpG sites for each DNA methylation prognostic subtype. The red bars and blue bars represent hypermethylation CpG sites and hypomethylation CpG sites, respectively. **b** The heatmap for the specific CpG sites in the 7 DNA methylation clusters. **c** The heatmap for specific methylation site annotated genes

To find the specific DNA methylation, CpGs were specifically hypermethylated or hypomethylated within particular RCC subgroups. We used EpiDiff software to identify the specific methylation sites and the QDMR software to quantify the methylation difference. Eventually, 437 of 3389 methylation sites were identified as cluster specific methylation sites. Specific hyper/hypomethylation CpG sites for each DNA methylation cluster are represented in Fig. 4a, which depicts cluster 4 having the largest number of specific methylations CpGs, most of which are hypomethylated. While others have a small number of specific methylations CpGs, most of them are hypermethylated. Nevertheless, no specific methylation CpGs was detected in cluster 6. Furthermore, the heatmap corresponding to the 437 specific methylation sites was constructed (Fig. 4b). The TNM classification, pathological grade and clinical stage, corresponding to 7 clusters of 437 specific methylation sites were evidently different, which suggested these were DNA methylation markers for the different subgroups in RCC.

We annotated these specific methylation sites for gene annotation and 437 specific methylation sites corresponding to 477 genes were identified. Besides, we explored the gene expression within the particular DNA methylation subgroups. Finally, the expression values in 151 samples of the train set for 436 of the 477 genes were obtained. Further, the heatmap for the expression profile of specific methylation site annotated genes was depicted (Fig. 4c). It can be seen that there were different expression patterns of these subgroups at the standard of expression profile, which indicated the specificities of these genes were consistent with the DNA methylation level and the gene expression level.

### TF enrichment of specific methylation site annotated genes

**Fig. 5:**
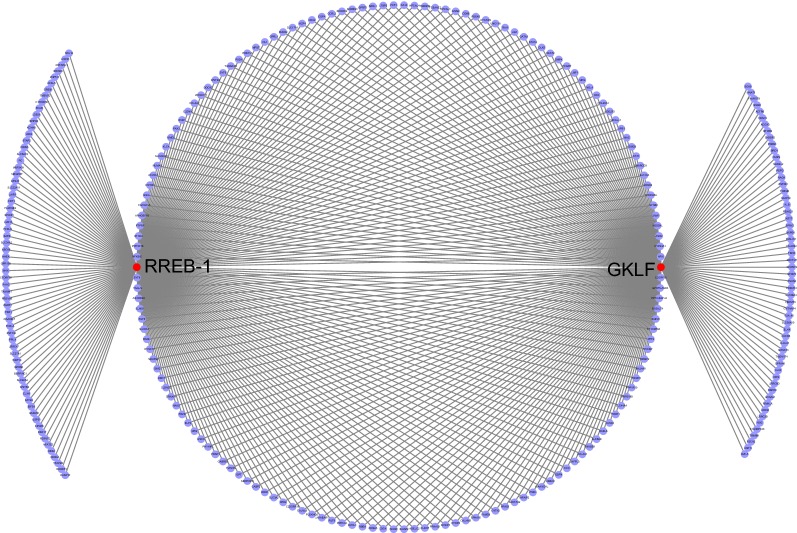
TF enrichment analysis of genes in the promoter methylation region. The red nodes and purple node represent TF and genes, respectively

To observe the mechanism of the 437 specific methylation sites, the genes, corresponding to the specific methylation sites in the promoter region were subjected to TF enrichment analysis. It was found that these genes were enriched with two transcription factors, GKLF and RREB-1 with significant false discovery rate (FDR) values of 0.00555 and 0.00173, respectively (Fig. 5). Besides, 277 genes were enriched with these two TFs, of which 212 and 221 were enriched with GKLF and RREB-1, respectively. Previously, many researchers have revealed that GKIF is a putative tumor suppressor gene epigenetically silenced in RCC by promoter CpG methylation [29–31]. These outcomes suggest that the genes that correspond to the 437 specific methylation sites in the promoter region of enrichment of GKLF and RREB-1, have potential values of prognosis and therapy for RCC.

### Validation of the prognosis prediction model

**Fig. 6:**
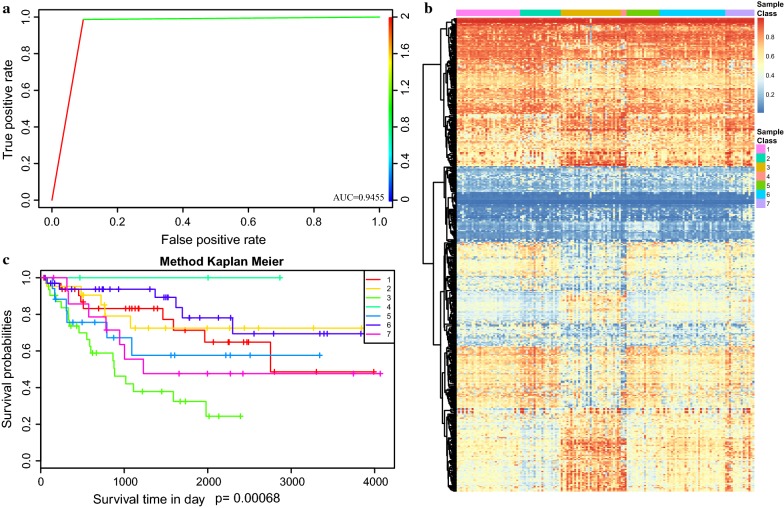
Construction and evaluation of the prognosis prediction model. **a** Area under roc (AUC) curve of the test set. **b** The heatmap for the specific CpG sites in the 7 DNA methylation clusters from test set. **c** Survival curves of 7 clusters predicted from the test set using the prognosis model

In order to verify the ability of discrimination of specific CpG sites for each prognosis subgroups in the set, we constructed a Bayesian classifier to determine the function of the prognosis prediction model, with 437 specific CpG sites as characters. The accuracy of the prognosis prediction model based on the train set was 81.7%. The area under the ROC curve reached 0.9455 (Fig. 6a). Then, we employed the prognosis model to predict cases in the test set. We extracted the expression profile data of 437 specific CpG sites from the test set and put them into a prognosis model for verification. The number of statistics in the test set was assigned to a similar class corresponding to the train set. Also, the heatmap corresponding to the 437 specific CpG sites in the test set was generated by the heatmap function using the prognosis model constructed from the train set. The results showed that there were also significant differences in the methylation patterns between the 7 categories of the prognosis prediction model, which was consistent with the train set (Fig. 6b). Moreover, the survival analysis of the 7 clusters in the test set showed that they were a significant prognostic difference (p < 0.00068) (Fig. 6c). The prognosis of cluster 2 was significantly better than the others, which is consistent with the train set.

**Fig. 7:**
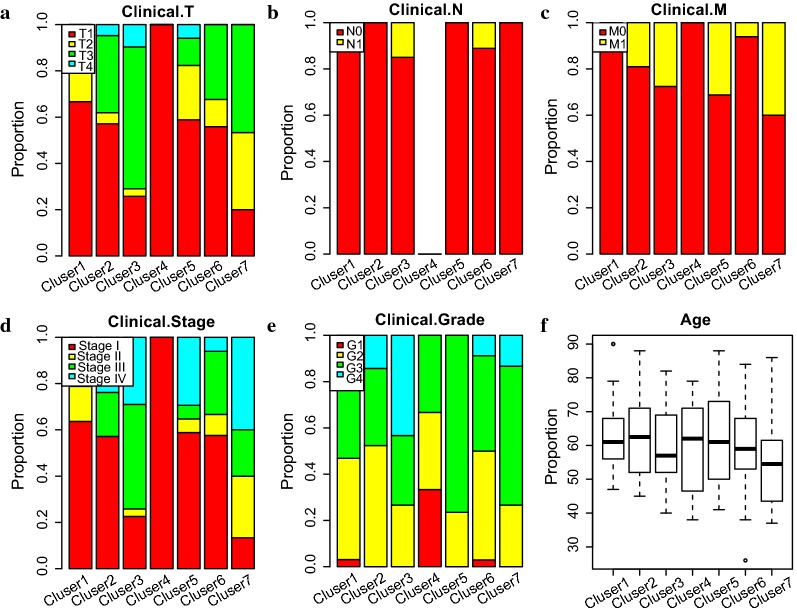
Characterization of different features of DNA methylation clustering from test set. **a** Proportion of different degrees of invasion in the 7 clusters. **b** Proportion of different degrees of lymphatic metastasis in the 7 clusters. **c** Proportion of different degrees of distant metastasis in the 7 clusters. **d** Proportion of clinical stage in the 7 clusters. **e** Proportion of pathological grade in the 7 clusters. **f** Age distribution in the 7 clusters

Next, we analyzed the distribution of TNM classification, pathological grade, clinical stage, and age in the prognosis of 7 molecular subtypes. A similar method as the train set was used and the relative consistent results were obtained (Fig. 7). In particular, clusters 1 and 4 had low invasiveness (Fig. 7a), clusters 3 and 6 had high relevance with lymphatic metastasis (Fig. 7b), clusters 3 and 7 had high relevance with distant metastasis (Fig. 7c), and clusters 3 and 7 connected with high pathological grade and high clinical stage (Fig. 7d, e). These results further illustrate the predictive accuracy of our model and the stability of its features.

**Fig. 8:**
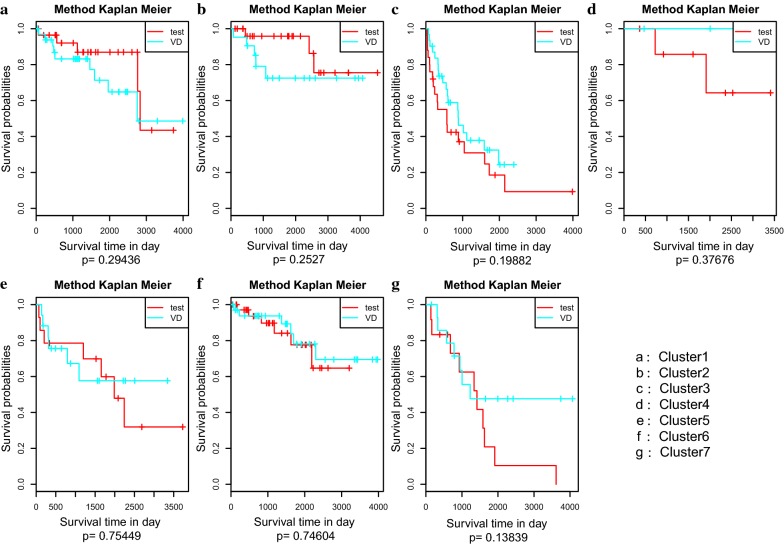
The survival curves of the same labelled clusters in the train set and test set

To examine whether the prognoses of clustering in the test set was similar to the corresponding clustering in the train set, we compared the prognostic relationship of 7 clusters between the test set and train set. There was no significant difference in the prognosis between the test set and the train set (Fig. 8). These results show that the cases classified or predicted to be in the same DNA methylation subgroups had the same prognosis.

### Comparison between 7 prognosis subgroups and 34-gene classifier (ClearCode34)

34-gene classifier (ClearCode34), which was a authoritatively subtyped predictor for classifying clear cell RCC according to good risk (ccA) and poor risk (ccB) subtype for assigning clear cell RCC patient and built a subtype-inclusive model to analyze patient survival outcomes according to risk stratification [9, 32]. The ClearCode34 classifier was applied for RNA-sequencing of data from 380 nonmetastatic clear cell RCC samples from TCGA. In the meantime, a total of 199 RCC samples in our study were selected to compare 7 prognosis subgroups and ClearCode34 classification results (Additional file 1: Table S1). From which it can be seen that C l and C 2 are mainly concentrated in ccA, whereas C 3, C 4, and C 7 mainly enriched in the ccB. ccA and ccB subtypes represented good prognosis and poor prognosis, respectively, which was consistent with our study that cluster 1 and cluster 2 have the best prognosis, whereas cluster 3 and cluster 7 have the worst prognosis (Fig. 3a). Interestingly, the ccA and ccB appeared in almost equal among patients in the C 6 subgroup, and therefore we compared the prognosis of ccA and ccB in the C 6 subgroup (Additional file 2: Figure S1). The results revealed that the prognosis of ccA was significantly better than the ccB, even for the C 6 subgroup in our study. Further, we compared the clinically important features of ccA and ccB in C 6, including TNM, stage, and grade. The findings revealed that there was significant difference in T 1 (p = 0.024), T 3 (*p *= 0.008), N 1 (*p *= 0.038), N X (*p *= 0.003), stage I (*p *= 0.024), and stage III (*p *= 0.008) between ccA and ccB in the C 6 subgroup (Additional file 3: Table S2). Moreover, most of the patients of ccA and ccB were distributed in T 1, N 0, stage 1, and grade 2, whereas less of the patients of ccA and ccB were distributed in T 2, N 1, stage 5, grade 1 in C 6 subgroup. The results were consistent with the analysis of the distribution of TNM classification, pathological grade, and clinical stage in the prognosis of 7 molecular subtypes (Fig. 3b, c, e, f). All these results demonstrate that our 7 prognosis subgroups and ClearCode34 classifier have a high degree of consistency with the RCC prognostic prediction and molecular classification.

## Discussion

Alterations in DNA methylation play an important role in the development and progression of RCC as confirmed in recent studies. In addition, the DNA methylated profiles provide insights into the etiology of RCC, especially for clinicians to provide clinically viable biomarkers for early diagnosis and precise treatment of RCC [33, 34]. Therefore, the whole genome DNA methylation spectrum sequencing technique has been widely used to study tumor-specific methylation alterations. Moreover, many different histology and benign adjacent RCC tissues of human or experimental animals have been extensively examined for DNA methylation for diagnosis and treatment. However, its effectiveness is limited due to factors such as high cost and heavy analytical burden and large sample size. Owing to the TCGA database, a publicly funded project has been prepared that aims at cataloging and discovering major carcinogenic genomic changes in more than 30 large cohorts of human tumors through large-scale genome sequencing and comprehensive multidimensional analysis [35]. In this study, 3389 significant CpG sites, corresponding to 307 RCC samples were obtained from TCGA for the classification analysis. Large sample sizes were used to provide an in-depth understanding of the etiology of RCC, supply proven biomarkers based on specific methylation of CpGs, and present a framework for the development of molecular subtypes based on DNA methylation for the detection of RCC patients.

RCC originates from the epithelial cells of the nephron, the functional unit of the kidney, and consists of several different molecular and histological subtypes. It has different clinical features, including therapeutic responses [36]. Therefore, it poses many challenges in the diagnosis and treatment of RCC, including the early diagnosis of malignant tumors, treatment selection, surgical benefits, side effects of chemotherapeutic drugs and prognosis assessment of patients. Since DNA methylation is one of the earliest molecular changes in cancer and is extensive and stable, its role in cancer biology has been extensively studied, including early diagnosis of renal cell carcinoma [37, 38]. Many studies have explored the role of DNA methylation in the etiology and progression of renal cell carcinoma. Clinical researchers have used DNA methylation as a biomarker for early diagnosis, prognosis and prediction, as well as the potential for DNA methylation to guide therapy [39, 40]. Therefore, we carried out this study to obtain a detailed classification of RCC epigenomes based on DNA methylation. Prognostic-related CpG sites were selected according to the classification of TNM, pathological grading, clinical staging, age, and survival analysis. In addition, according to the inherent molecular characteristics of DNA methylation, RCC can be divided into seven subtypes, which corresponds to the significant difference in clinical results and molecular subtypes. Thus, the classification scheme provided a molecular stratification suitable for individual tumors, which has the significance of influencing therapeutic decision-making and defined the biological mechanisms involved in the progression of RCC.

RCC is consists of a heterogeneous group of tumors, and according to the morphological and histological classification, RCC can be classified as clear cell carcinomas (60% of cases), papillary tumors, chromophobic tumors, oncocytomas, and collecting or Bellini duct tumors [41]. TNM stage and Fuhrman grade are still the most commonly used predictors of clinical prognosis in RCC patient. In addition, combined with comprehensive clinical systems such as the Mayo Clinic stage, size, grade and necrosis (SSIGN) score and the University of California Integrated Staging System, clinicians have improved the accuracy of prognosis in RCC patients [42]. However, RCC patients with different cell types originating from renal units, as well as their histological, molecular, genetic and clinical diversity, may have different results with similar clinical characteristics or comprehensive system scores. Therefore, accurate classification of RCC is crucial for prognostic risk stratification, targeted therapy selection, and gene detection and identification. Although the molecular profiling of RCC subtypes has been identified as a unique and diverse spectrum of changes that are used to improve our prognosis and therapeutic effects in the initial stage, their development has been rapid [43, 44]. In this study, we used significant CpG sites to identify 7 distinct prognostic subgroups of RCC that seemed to predict disease-specific survival, as well as TNM classification and pathological grade, clinical stages, and age distribution of prognosis of 7 molecular subtypes. Therefore, the classification scheme provided a molecular stratification suitable for individual tumors, which significantly influenced treatment decision-making and accurate diagnosis. For example, an RCC patient was diagnosed as RCC at T1, which meant a primary tumor was limited to kidney, maximum diameter ≤ 7 cm. Based on the 7 molecular subtypes, when the significant CpG sites of patients were assigned to cluster 7, the patients were found to have high chances of developing lymph nodes, distant metastasis and had poor prognosis. This is necessary for early radical resection of RCC, to assess whether early lymph node dissection is needed to prevent metastasis. Whereas, when the significant CpG sites of patients were assigned to cluster 1, the tumors exhibited low degree of invasion and predicted a good prognosis. This result can prompt clinicians to re-evaluate the treatment of patients, whether to have radical total RCC or partial resection or close clinical follow-up, or chemotherapeutic therapy, which can help alleviate pain and benefit patients. In conclusion, our research on the 7 subgroups at the level of DNA methylation molecular can classify RCC more accurately and guide clinicians when diagnosing, treating and performing prognosis of different epigenetic subtypes.

Previous researches reported that CpG island methylation as an epigenetic form of gene regulation that disrupts the function of tumor suppressor genes or oncogenes and was shown to promote carcinogenesis [45]. Based on the DNA methylation and gene expression profiles in the tumor, we found 437 specific methylation sites corresponding to 477 genes, and they defined the particular DNA methylation subgroups of RCC. Besides, we found that the levels of DNA methylation and gene expression based on 7 subgroups were consistent, which suggested that the changes in methylation in some regions might be associated with RCC by changing the expression of corresponding genes. Moreover, a prognostic model characterized by 437 specific CpGs could be used to divide the test group into different prognostic clusters, which was consistent with the results of the classification. Hypomethylation and hypermethylation of DNA can affect the progression and prognosis of RCC, leading to overexpression of oncogenes and down-regulation of tumor suppressor genes, respectively [46]. On the other hand, abnormal DNA methylation was significantly associated with poor tumor differentiation, tumor invasion and poor prognosis in cancer patients [47]. In our study, we found that cluster 1 had a small number of specific methylations CpGs and cluster 6 had no specific methylation CpGs, which is a reasonable explanation of clusters 1 and 6 having low invasiveness. Additionally, this might explain the reason for the cluster 1 having the best prognosis on survival analysis.

Increasing evidence has shown that epigenetic regulation, such as abnormal hypermethylation of CpG islands in promoters, is key to tumorigenesis [48]. In our study, the genes, corresponding to 437 specific methylation sites in the promoter region were considered for TF enrichment analysis. We found that the genes in the promoter methylation were enriched with TF of GKLF and RREB-1. Li et al. found that significant reduction or loss of GKLF in RCC was mainly due to promoter methylation abnormalities and that GKLF deletion was associated with the progression of RCC, relatively low overall survival, and disease-free survival [29]. Liu et al. found that RREB-1 regulated the transcription of the p53 gene, which is a tumor suppressor gene, through the core promoter element of p53 after genotoxic stress. The silencing of RREB-1 significantly lowers the expression of p53 mRNA and protein, which can lead to tumorigenesis [49]. Even though there is less literature focusing on the relationship between RCC and RREB-1, some mechanistic studies have suggested that the RREB-1 has a major role in tumor suppression of pancreatic cancer [50], bladder cancer [51], prostate cancer [52]. These outcomes imply that TFs of GKLF and RREB-1 promotes methylation in RCC biomarkers recognition, and highlights their diagnostic and prognostic values, and clinical application.

## Conclusion

This study identified a new classification of RCC into 7 prognosis subgroups according to the DNA methylation data, indicating that molecular subtypes are independent prognostic factors of RCC. This provides a more detailed explanation of molecular subtypes, as a simple pathological classification. In addition, this classification will help to identify new biomarkers of RCC and provide more accurate subdivision of RCC. Changes in DNA methylation can be used as markers of molecular profiles associated with standard clinical features, which could significantly enhance the diagnosis of RCC and facilitate individualized treatment for patients. Furthermore, the specific CpG sites and corresponding genes in specific subgroups can be used as biomarkers for early diagnosis, accurate treatment and precise prognosis prediction. Finally, the framework proposed in this work can be used to study new classifications of molecular subtypes associated with specific tumors.

## Additional files

**Additional file 1: Table S1.** The distribution of 199 RCC samples based on 7 prognosis subgroups and ClearCode34 classification.

**Additional file 2: Figure S1.** Prognostic difference between ccA and ccB in C 6 subgroup.

**Additional file 3: Table S2.** Analysis of clinical features of ccA and ccB in C 6 subgroup.

## Data Availability

The datasets generated and analysed during the current study are available in the The Cancer Genome Atlas database.

## Abbreviations

RCC: renal cell carcinoma
WHO: World Health Organization
5Mc: 5-methyldeoxycytidine
TCGA: The Cancer Genome Atlas
KNN: k-nearest neighbours
QDMR: quantitative differentially methylated region
DMRs: differentially methylated regions
ROC: receiver operating characteristic curve
