# Supplementary material for: DNA methylation-based classification and identification of renal cell carcinoma prognosis-subgroups
Source: Cancer Cell Int. 2019 Jul 16;19:185. doi: 10.1186/s12935-019-0900-4 (PMC6636124; doi:10.1186/s12935-019-0900-4)
Supplement: Supplementary file 1 — Additional file 1: Table S1. The distribution of 199 RCC samples based on 7 prognosis subgroups and ClearCode34 classification. [file 12935_2019_900_MOESM1_ESM.docx]

Table 2. The distribution of 199 RCC samples based on 7 prognosis subgroups and ClearCode34 classification.

|  | ccA | ccB |
| --- | --- | --- |
| C1 | 19 | 1 |
| C2 | 14 | 1 |
| C3 | 2 | 12 |
| C4 | 1 | 6 |
| C5 | 6 | 1 |
| C6 | 64 | 65 |
| C7 | 2 | 5 |
