# Supplementary figures and images for: DNA methylation-based classification and identification of renal cell carcinoma prognosis-subgroups
Source: Cancer Cell Int. 2019 Jul 16;19:185. doi: 10.1186/s12935-019-0900-4 (PMC6636124; doi:10.1186/s12935-019-0900-4)

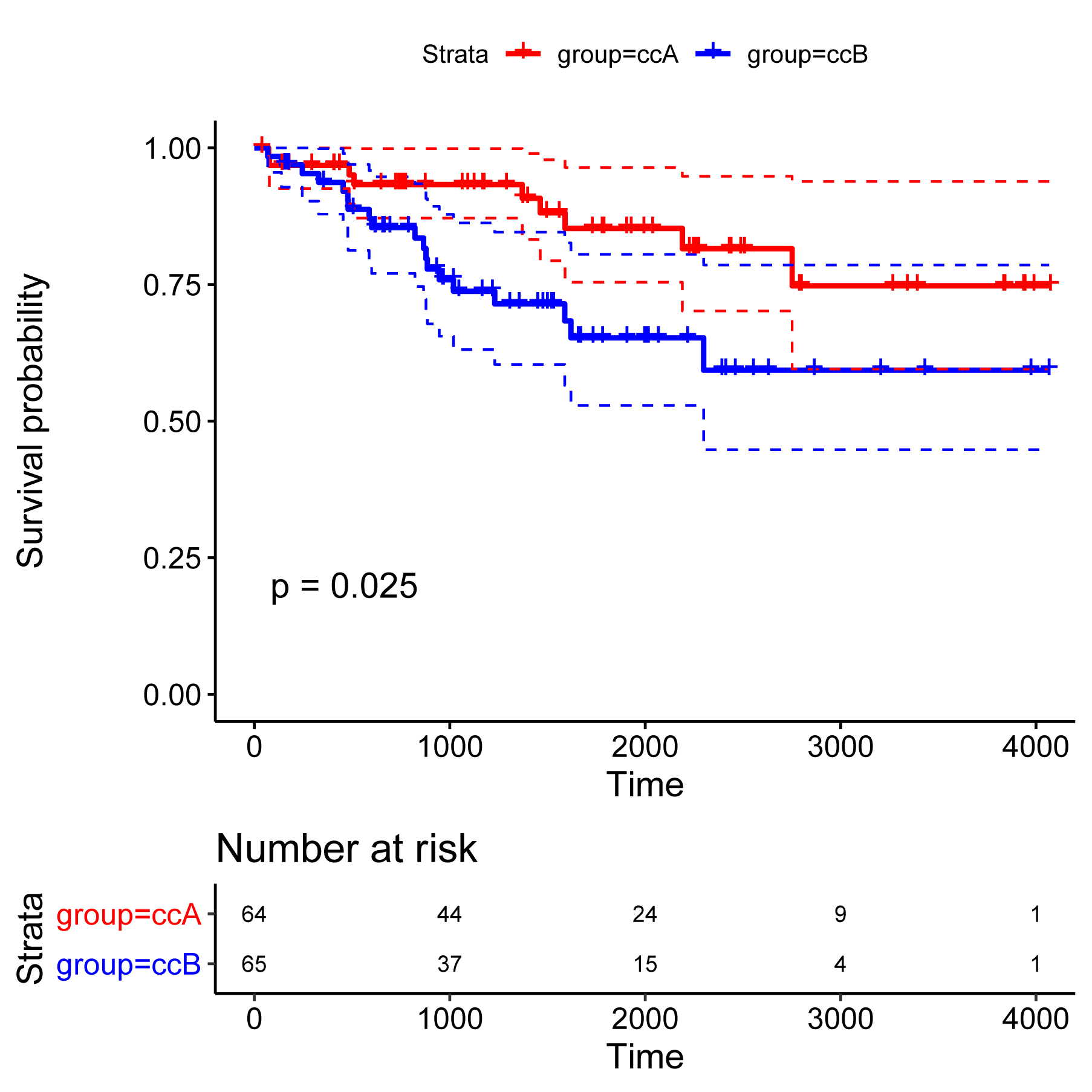

Supplement: Supplementary file 2 — Additional file 2: Figure S1. Prognostic difference between ccA and ccB in C 6 subgroup. [file 12935_2019_900_MOESM2_ESM.tif]
