# Supplementary material for: DNA methylation-based classification and identification of renal cell carcinoma prognosis-subgroups
Source: Cancer Cell Int. 2019 Jul 16;19:185. doi: 10.1186/s12935-019-0900-4 (PMC6636124; doi:10.1186/s12935-019-0900-4)
Supplement: Supplementary file 3 — Additional file 3: Table S2. Analysis of clinical features of ccA and ccB in C 6 subgroup. [file 12935_2019_900_MOESM3_ESM.docx]

Table 3. Analysis of clinical features of ccA and ccB in C 6 subgroup.

|  | ccA | ccB | *P* value |
| --- | --- | --- | --- |
| T1 | 44 | 31 | 0.024737331 |
| T2 | 7 | 6 | 0.976485375 |
| T3 | 12 | 27 | 0.00863849 |
| T4 | 1 | 1 | 1 |
| N0 | 23 | 35 | 0.061848274 |
| N1 | 0 | 6 | 0.038353996 |
| NX | 41 | 24 | 0.003656778 |
| M0 | 64 | 65 | － |
| M1 | － | － | － |
| Stage I | 44 | 31 | 0.024737331 |
| Stage II | 7 | 5 | 0.740399271 |
| Stage III | 12 | 27 | 0.00863849 |
| Stage IV | 1 | 1 | 1 |
| G1 | 2 | 0 | 0.469235547 |
| G2 | 33 | 29 | 0.539623541 |
| G3 | 25 | 23 | 0.802637057 |
| G4 | 4 | 12 | 0.066251462 |
| GX | 0 | 1 | 1 |
